# Treatment patterns of patients with migraine eligible for anti-CGRP pathway monoclonal antibodies

**DOI:** 10.3389/fneur.2024.1433423

**Published:** 2024-08-06

**Authors:** Ani C. Khodavirdi, Jasjit K. Multani, Sam S. Oh, Fiston Vuvu, Mark E. Bensink, Karen M. Stockl, Kevin Hawkins, Chia-Chun Chiang, A. Laine Green, Stewart J. Tepper

**Affiliations:** ^1^Amgen Inc., Thousand Oaks, CA, United States; ^2^IQVIA, Plymouth, PA, United States; ^3^Benofit Consulting, Brisbane, QLD, Australia; ^4^Mayo Clinic, Rochester, MN, United States; ^5^Mayo Clinic, Scottsdale, AZ, United States; ^6^The New England Institute for Neurology and Headache, Stamford, CT, United States

**Keywords:** chronic migraine, anti-calcitonin gene-related peptide, treatment patterns, non-chronic migraine, monthly headache days

## Abstract

**Introduction:**

Migraine is a debilitating neurological disorder, with a wide range of symptoms and disease burden, underscoring the heterogeneity of patients’ disease characteristics and treatment needs. To characterize the profile of migraine patients in the US who may be eligible for preventive treatment with an anti-CGRP pathway mAb and to better understand treatment patterns and real-world use of acute and preventive medications for migraine, we conducted a retrospective cohort study of adult patients.

**Methods:**

These patients were identified as having migraine using diagnosis codes or migraine-specific medication use (first = index) in the IQVIA PharMetrics® Plus database. Patients were required to have ≥ 12 months of continuous enrollment in medical and pharmacy benefits prior to index (baseline) and after index (follow-up). Patients were stratified into chronic migraine (CM) and non-chronic migraine (non-CM) by diagnosis codes. Based on acute migraine-specific medication dispensing data in the follow-up period, non-CM patients were divided into 3 cohorts: highest, middle, and lowest tertile of total units of dispensed acute migraine-specific medication (gepants, ditans, ergot derivatives, and triptans). Migraine medication use was captured in the baseline and follow-up periods.

**Results:**

A total of 22,584 CM and 216,807 non-CM patients (72,269 patients in each tertile) were identified and included in the study. Over the follow-up, CM patients had a mean of 70 units of acute migraine-specific medications dispensed, while the highest, middle, and lowest tertile of non-CM patients had a mean of 92, 29, and 10 units, respectively. Anti-calcitonin gene-related peptide pathway mAbs were dispensed for 28.9% of CM patients, and for 6.9%, 4.1%, and 2.9% of non-CM patients in the highest, middle, and lowest tertiles, respectively.

**Conclusion:**

A lower proportion of non-CM patients had use of anti-calcitonin gene-related peptide pathway mAbs compared to CM patients, confirming the unmet need with appropriate preventive medication. There appears to be a persistent gap in management of patients without a diagnosis of CM who are dispensed high quantities of acute migraine-specific medications.

## 1 Introduction

Migraine is a chronic neurological disorder causing headaches with a multitude of debilitating symptoms, significantly impacting an individual’s daily functioning and quality of life (1–4). Migraine impacts approximately 11% of men in the United States (US) and 21% of women (5, 6). Symptoms may include visual disturbances, pulsating pain, nausea, photophobia, and speech and cognitive changes, and may occur before, during, or after a migraine attack (7–9).

Currently, migraine is divided into chronic and episodic, with CM defined as ≥15 monthly headache days (MHDs) for ≥3 months with at least eight migraine days per the third edition of the International Classification of Headache Disorders (ICHD-3) (10). Albeit not defined by ICHD-3, episodic migraine (EM) has been described as 14 or fewer headache days per month.

Studies examining patients with EM have demonstrated a wide range of symptom severity and disability that may change from month to month, underscoring the heterogeneity of this group and further challenging the 15 MHD threshold as an accurate reflection of disease burden and treatment needs (5, 11, 12). Due to these variations, particularly that of patient disease burden, determining and subsequently optimizing therapy can be arduous and lengthy, with considerable burden on the patient and the healthcare system to find the most appropriate treatment option (13, 14). Thus, studies evaluating patients with EM might provide a better understanding of patient needs and provide useful clinical information about their treatment patterns if patients were stratified by the severity of their migraine and associated symptoms.

Acute treatments are used to relieve pain and other associated symptoms during a migraine attack (15). While effective in many patients, they may be contraindicated and/or not tolerated in others, limiting their utility (4). Patients who experience frequent migraine attacks tend to use higher quantities of acute medications. With traditional acute medications, medication overuse, defined as use on ≥10 or ≥15 days per month, depending on the different types of medications being used, can lead to worsening of the existing headache condition or the development of medication overuse headache (MOH) (16, 17). In a survey completed as part of the American Migraine Prevalence and Prevention (AMPP) study, overuse of barbiturates and any use of opiates were found to be associated with the development of CM in patients formerly without CM (17). Optimizing migraine preventive treatments is an important priority in reducing reliance on acute medications (18, 19).

A persistent challenge in migraine management with traditional oral migraine preventive medications has been poor adherence, even among patients with a CM diagnosis. One retrospective study of CM patients observed 50% discontinuation rate within 2 months of treatment initiation and an overall persistence of 25% at 6 months (14). Treatment discontinuation, in general, and also applicable to traditional migraine preventive medications, is most often due to a lack of efficacy, poor tolerability, and the occurrence of side effects (20, 21).

The discovery of the calcitonin gene-related peptide (CGRP) pathway’s significant role in migraine pathophysiology has led to the development of the very first migraine-specific class of drugs (21). This class includes monoclonal antibodies (erenumab, galcanezumab, fremanezumab, and eptinezumab) approved for migraine prevention in the US (13) and gepants indicated for prevention (atogepant and rimegepant) and acute use (rimegepant, ubrogepant, and zavegepant). The American Headache Society (AHS) published a position statement in April 2024 that anti-CGRP pathway therapies should be the first line for migraine prevention rather than older, non-specific preventive medications (22). The current study was undertaken to characterize the profile of migraine patients in the US who may be eligible for preventive treatment with an anti-CGRP pathway mAb and to better understand treatment patterns and the real-world use of acute and preventive medications for migraine.

## 2 Materials and methods

### 2.1 Study design

We conducted a descriptive, retrospective cohort study using the IQVIA PharMetrics® Plus database, with a study period of 1 January 2018 to 31 December 2020. IQVIA PharMetrics® Plus is a health plan claims database comprised of fully adjudicated medical and pharmacy claims for more than 190 million unique enrollees since 2006. Data contributors to the database are largely commercial health plans, and the database is representative of the commercially insured US national population for patients under 65 years of age. All data are compliant with the Health Insurance Portability and Accountability Act (HIPAA) to protect patient privacy.

### 2.2 Patient population

The identification period for patients with migraine was from 1 January 2019 to 31 December 2019. Patients were included based on either of two criteria: ≥1 non-ancillary claim with an ICD-10-CM diagnosis code for migraine (G43.x) (i.e., patients with CM) or ≥1 claim for migraine-specific treatments (anti-CGRP pathway mAbs, gepants, ditans, ergot derivatives, and triptans) (i.e., patients with migraine but not CM), identified by NDC codes, in the study time period. For the purposes of this study and since a diagnosis code for EM does not exist, patients without a CM diagnosis but with a claim for migraine-specific treatments have been categorized as non-CM. An ancillary claim is diagnostic where the result may not indicate the patient has the disease; therefore, we do not include these claims to ensure confidence in diagnoses captured.

The date of the first qualifying claim was considered the index date. To be included for analysis, patients were further required to be between the ages of 18 and 64 years on the index date and have 12 months of continuous enrollment (an indication of medical and pharmacy coverage) in the database prior to and after the index date, where 1 month is equal to 30 days. The 12 months prior to the index date were considered the baseline period, and the 12 months after, including the index date, were considered the follow-up period. Patients with data quality issues, such as missing age or gender, were excluded from the study. Patients with a diagnosis of cluster headache (G44.00x, G44.01x, and G44.02x) were also excluded, as most treatments of interest to this study are not indicated for cluster headache (15). Patients identified by diagnosis code were required to have ≥1 claim for migraine-specific treatment in the follow-up period.

### 2.3 Analysis

Migraine patients were stratified into a total of four cohorts, based first on diagnosis code; patients with diagnosis codes for CM (G43.7) on the index date or during the 12-month baseline period were considered CM patients. Patients without a diagnosis for CM and with dispensed AMSM during follow-up, where AMSM use was defined as a prescription for triptans, ergot derivatives, gepants, or ditans, were considered non-CM patients. The non-CM patients were then stratified into three tertiles of equal size (highest, middle, and lowest) based on the number of AMSM units dispensed. Tertiles were chosen to correspond with the sub-classification of patients to low-, moderate-, and high-frequency non-CM, as described in Lipton et al. (5) Stratification cutoffs were data-driven and not determined prior to analysis. Dispensed units were drawn from the outpatient pharmacy claims; each claim contains information on the unit of medication, which includes a number of pills, tablets, or milliliters of a liquid oral or injectable medication.

Demographic variables such as age, gender, and geographic region were measured on the index date, and clinical characteristics, namely comorbidities and treatments, were captured in the baseline period. Comorbidities were captured using ICD-10-CM diagnosis and procedure codes, and treatments were captured using NDC codes and, where applicable, HCPCS codes, with treatments including traditional acute and preventive medications for migraine as well as anti-CGRP pathway mAbs. Of the patients with a provider visit in the follow-up, the provider specialty was captured and presented as the proportion of the total patient cohort for each specialty. Findings from this study are reported descriptively, with no statistical comparisons between cohorts.

## 3 Results

From January to December 2019, we identified just over 360,000 patients with a migraine diagnosis claim and approximately 1.3 million patients with a claim for AMSMs (patients with both a diagnosis claim for migraine and a claim for a migraine treatment on the index date were counted as indexing with migraine diagnosis). After applying inclusion and exclusion criteria, 239,391 patients were eligible for analysis. Of these, less than 10% (9.4%, *N* = 22,584) had a CM diagnosis on an index or in the baseline period; the remaining 90.6% of patients were considered non-CM and divided into 3 tertiles composed of 72,269 patients each. In the follow-up period, the mean dispensed units of AMSM for CM were 70 units (median: 54); in the highest, middle, and lowest tertiles, the mean of dispensed AMSM was 92 (median: 81), 29 (median: 27), and 10 (median: 9), respectively.

### 3.1 Patient characteristics

Non-CM patients with the highest AMSM quantity dispensed were older (mean: 47 years) and had a higher proportion of females (85.3%) compared to non-CM patients with middle and lowest AMSM quantity dispensed (mean of 44 and 42 years; 84.8 and 80.8%, respectively). CM patients had a mean age of 45 years, with 89% of them being female. Nearly all patients had commercial insurance coverage.

The most common comorbidities seen across cohorts were anxiety (non-CM: 23.6–24.7%; CM: 35.2%), depression (non-CM: 16.9–18.8%; CM: 28.0%), and hypertension (non-CM: 18.8–19.9%; CM: 22.3%); see Table 1 for full demographic and clinical characteristics for all cohorts.

**Table 1:** Patient demographics and clinical characteristics, in CM and non-CM cohorts.

	Chronic migraine *N* = 22,584	Non-CM *N* = 216,807		
		Highest tertile of AMSM quantity dispensed^1^ (*N* = 72,269)	Middle tertile of AMSM quantity dispensed^1^ (*N* = 72,269)	Lowest tertile of AMSM quantity dispensed^1^ (*N* = 72,269)
**Age**				
Mean (SD)	45.4 (11.0)	47.3 (10.6)	44.4 (11.9)	41.6 (12.6)
Median	47	49	46	42
**Sex, *n* (%)**				
Female	20,094 (89.0%)	61,654 (85.3%)	61,266 (84.8%)	58,358 (80.8%)
Male	2,490 (11.0%)	10,615 (14.7%)	11,003 (15.2%)	13,911 (19.2%)
**Geographic region, *n* (%)**				
Northeast	4,096 (18.1%)	13,105 (18.1%)	12,543 (17.4%)	12,551 (17.4%)
Midwest	6,090 (27.0%)	19,534 (27.0%)	19,989 (27.7%)	19,359 (26.8%)
South	9,602 (42.5%)	31,258 (43.3%)	30,891 (42.7%)	31,220 (43.2%)
West	2,796 (12.4%)	8,372 (11.6%)	8,846 (12.2%)	9,139 (12.6%)
**Insurance type, *n* (%)**				
Commercial/self-insured	22,156 (98.1%)	71,400 (98.8%)	71,386 (98.8%)	71,400 (98.8%)
Medicare advantage	284 (1.3%)	490 (0.7%)	440 (0.6%)	401 (0.6%)
Medicaid	144 (0.6%)	379 (0.5%)	443 (0.6%)	468 (0.6%)
**Most frequent comorbid conditions, *n* (%)**				
Asthma	2,730 (12.1%)	5,648 (7.8%)	6,314 (8.7%)	6,361 (8.8%)
Autoimmune	1,541 (6.8%)	3,712 (5.1%)	3,443 (4.8%)	3,199 (4.4%)
Rheumatoid arthritis	526 (2.3%)	1,330 (1.8%)	1,093 (1.5%)	958 (1.3%)
Psoriasis	334 (1.5%)	882 (1.2%)	884 (1.2%)	827 (1.1%)
Systemic lupus erythematosus	261 (1.2%)	492 (0.7%)	475 (0.7%)	460 (0.6%)
Psoriatic arthritis	141 (0.6%)	360 (0.5%)	313 (0.4%)	291 (0.4%)
Autoimmune thyroiditis	519 (2.3%)	1,144 (1.6%)	1,099 (1.5%)	1,043 (1.4%)
Cardiovascular disease	5,157 (22.8%)	14,414 (19.9%)	14,640 (20.3%)	13,875 (19.2%)
Hypertension	5,040 (22.3%)	14,106 (19.5%)	14,361 (19.9%)	13,589 (18.8%)
Angina, stable	127 (0.6%)	253 (0.4%)	263 (0.4%)	299 (0.4%)
Angina, unstable	48 (0.2%)	123 (0.2%)	148 (0.2%)	139 (0.2%)
Heart failure	104 (0.5%)	326 (0.5%)	333 (0.5%)	313 (0.4%)
Acute myocardial infarction	88 (0.4%)	209 (0.3%)	227 (0.3%)	235 (0.3%)
Ventricular arrhythmia	37 (0.2%)	102 (0.1%)	86 (0.1%)	85 (0.1%)
Cerebrovascular disease	313 (1.4%)	382 (0.5%)	488 (0.7%)	600 (0.8%)
Ischemic stroke	165 (0.7%)	206 (0.3%)	245 (0.3%)	319 (0.4%)
Hemorrhagic stroke	28 (0.1%)	34 (0.1%)	44 (0.1%)	55 (0.1%)
Transient ischemic attack	177 (0.8%)	198 (0.3%)	289 (0.4%)	341 (0.5%)
Gastrointestinal	3,673 (16.3%)	6,881 (9.5%)	6,864 (9.5%)	6,487 (9.0%)
Constipation	1,919 (8.5%)	3,259 (4.5%)	3,250 (4.5%)	3,164 (4.4%)
Irritable bowel syndrome	1,513 (6.7%)	2,488 (3.4%)	2,547 (3.5%)	2,281 (3.2%)
GI Bleed	626 (2.8%)	1,288 (1.8%)	1,351 (1.9%)	1,346 (1.9%)
Crohn’s disease	185 (0.8%)	501 (0.7%)	446 (0.6%)	415 (0.6%)
Ulcerative colitis	210 (0.9%)	510 (0.7%)	484 (0.6%)	395 (0.6%)
Non-cerebrovascular central nervous system	10,196 (45.2%)	23,663 (32.7%)	23,123 (32.0%)	21,998 (30.4%)
Anxiety	7,955 (35.2%)	17,815 (24.7%)	17,595 (24.4%)	17,023 (23.6%)
Depression	6,325 (28.0%)	13,571 (18.8%)	13,222 (18.3%)	12,192 (16.9%)

1 The sum of the acute migraine medication quantities dispensed during the follow-up period was used to derive the tertiles of AMSM use (triptans, ergot derivatives, gepants, or ditans).

### 3.2 Treatment patterns

In the baseline period, all CM patients had claims for an AMSM compared to 58% of the lowest tertile non-CM patients and 67% of the highest tertile non-CM patients. The vast majority of CM patients had a claim for a triptan (87.1%), and a sizeable proportion had claims for opioids and non-steroidal anti-inflammatory drugs (NSAIDs) (39.7 and 40.3%, respectively), with 64.7% using non-triptans overall. Baseline period triptan utilization differed for the highest tertile non-CM patients, who had similar utilization rates (82.3%) as CM patients. Middle and lowest tertiles of AMSM dispensed had lesser utilization of triptans: 55.7 and 27.6%, respectively. Utilization rates of non-triptans were similar between tertiles of acute medication dispensed, from 42.0% in the lowest tertile of acute medication dispensed to 46.9% in the highest tertile of acute medication dispensed. We did not identify any CM or non-CM patients with a claim for a gepant in the baseline period (Table 2).

**Table 2:** Migraine treatments in the baseline and follow-up period.

	Chronic migraine^1^ *N* = 22,584		Non-CM *N* = 216,807					
			Highest tertile of AMSM quantity dispensed^2^ (*N* = 72,269)		Middle tertile of AMSM quantity dispensed^2^ (*N* = 72,269)		Lowest tertile of AMSM quantity dispensed^2^ (*N* = 72,269)	
	Baseline	Follow-up	Baseline	Follow-up	Baseline	Follow-up	Baseline	Follow-up
**Traditional acute anti-migraine agents (*n*, %)**								
Any acute agent	22,584 (100.0%)	22,584 (100.0%)	48,068 (66.5%)	72,269 (100.0%)	44,584 (61.7%)	72,269 (100.0%)	42,112 (52.3%)	72,269 (100.0%)
Triptans	19,660 (87.1%)	22,254 (98.5%)	59,471 (82.3%)	72,120 (99.8%)	40,224 (55.7%)	71,989 (99.6%)	19,969 (27.6%)	71,700 (99.2%)
Ergot derivatives	563 (2.5%)	627 (2.8%)	183 (0.3%)	399 (0.6%)	125 (0.2%)	322 (0.4%)	127 (0.2%)	439 (0.6%)
Opioids	8,975 (39.7%)	8,422 (37.3%)	21,658 (30.0%)	20,912 (29.0%)	19,567 (27.1%)	18,992 (26.3%)	18,117 (25.1%)	17,786 (24.6%)
NSAIDs	9,103 (40.3%)	8,859 (39.2%)	20,013 (27.7%)	21,006 (29.1%)	19,772 (27.4%)	21,174 (29.3%)	19,074 (26.4%)	20,981 (29.0%)
Non-NSAID/non-opioid analgesics^3^	4,050 (17.9%)	3,672 (16.3%)	6,214 (8.6%)	6,490 (9.0%)	5,120 (7.1%)	5,979 (8.3%)	4,794 (6.6%)	6,265 (8.7%)
Lasmiditan	0 (0.0%)	4 (0.02%)	0 (0.0%)	13 (0.02%)	0 (0.0%)	5 (0.01%)	0 (0.0%)	10 (0.01%)
Rimegepant	0 (0.0%)	7 (0.01%)	0 (0.0%)	65 (0.09%)	0 (0.0%)	65 (0.09%)	0 (0.0%)	63 (0.09%)
Ubrogepant	0 (0.0%)	47 (0.2%)	0 (0.0%)	168 (0.23%)	0 (0.0%)	253 (0.4%)	0 (0.0%)	128 (0.2%)
**Traditional preventive anti-migraine agents (*n*, %)**								
Any preventive agent	18,778 (83.2%)	17,980 (79.6%)	41,488 (57.4%)	45,395 (62.8%)	36,715 (50.8%)	42,291 (58.5%)	32,545 (45.0%)	39,054 (54.0%)
Select anticonvulsant medication use^4^	11,603 (51.4%)	10,318 (45.7%)	18,185 (25.2%)	20,864 (28.9%)	14,159 (19.6%)	17,886 (24.7%)	11,623 (16.1%)	15,648 (21.7%)
Select antihypertensive medication use^5^	7,342 (32.5%)	6,968 (30.9%)	14,833 (20.5%)	17,306 (23.9%)	12,655 (17.5%)	15,704 (21.7%)	11,040 (15.3%)	13,855 (19.2%)
Select antidepressant medication use^6^	11,615 (51.4%)	11,179 (49.5%)	25,164 (34.8%)	27,649 (38.3%)	22,714 (31.4%)	25,969 (35.9%)	20,043 (27.7%)	23,588 (32.6%)
Select botulinum toxin medication use^7^	1,145 (5.1%)	1,099 (4.9%)	99 (0.1%)	221 (0.3%)	51 (0.01%)	124 (0.2%)	42 (0.1%)	125 (0.2%)
Other medications that prevent migraines^8^	3,456 (15.3%)	3,477 (15.4%)	4,782 (6.6%)	5,403 (7.5%)	3,738 (5.2%)	4,448 (6.2%)	3,228 (4.5%)	3,827 (5.3%)
CGRP mAbs^9^	2,297 (10.2%)	6,536 (28.9%)	589 (0.8%)	4,968 (6.9%)	242 (0.3%)	2,983 (4.1%)	192 (0.3%)	2,129 (2.9%)

1 Patients with ICD-10-CM G43.7 or G43.7x diagnosis code during the 12-month post-index period (including index date) are considered to have chronic migraine.

2 Acute medication use frequency is defined by the sum of the quantity dispensed during the post-index period for any of the following acute medications: (1) triptans, (2) ergot derivatives, or (3) gepants or ditans.

3 Non-NSAID/non-opioid analgesics are defined as acetaminophen, baclofen, butalbital, and ziconotide.

4 Select anticonvulsant medications include oral formulations of carbamazepine, gabapentin, levetiracetam, pregabalin, topiramate, valproate sodium, valproic acid, divalproex sodium, and zonisamide.

5 Select antihypertensive medications include oral formulations (unless noted otherwise) of atenolol, bisoprolol, metoprolol, nadolol, nebivolol, pindolol, propranolol, timolol, verapamil, candesartan, clonidine (oral and transdermal patch formulations), lisinopril, and olmesartan medoxomil.

6 Select antidepressant medications include oral formulations of duloxetine, desvenlafaxine, venlafaxine, amitriptyline, desipramine, doxepin, imipramine, nortriptyline, protriptyline, clomipramine, escitalopram, citalopram, sertraline, and mirtazapine.

7 Select botulinum toxin medications include abobotulinumtoxinA injection, incobotulinumtoxinA injection, onabotulinumtoxinA injection, and rimabotulinumtoxinB injection. These medications are identified with NDCs on prescription claims and HCPCS on medical claims.

8 Other medications that prevent migraines include oral formulations of carisoprodol, cyproheptadine, guanfacine, memantine, milnacipran, and tizanidine.

9 CGRP mAbs include erenumab, galcanezumab, fremanezumab, and eptinezumab.

**Figure 1:**
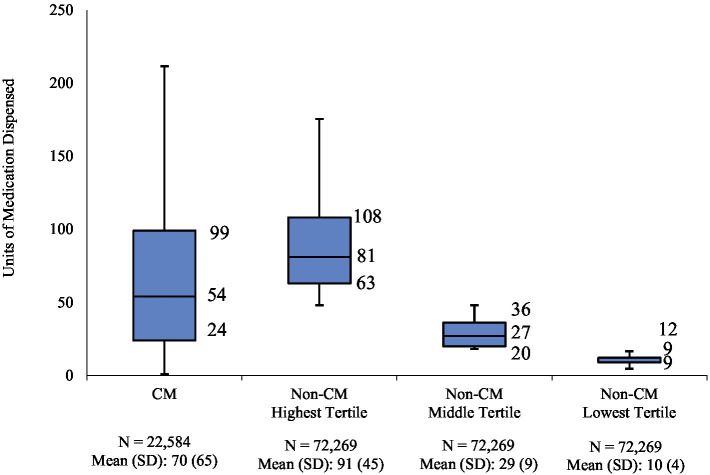
Distribution of dispensed quantity (units) of acute migraine medications in the follow-up period. Acute migraine medications (triptans, ergot derivatives, gepants, or ditans) utilized over the 12-month follow-up were evaluated for each patient; non-CM patients were split into three equal cohorts (tertiles of lowest, medium, and highest usage) based on medication utilization levels; numbers represent 25th, 50th, and 75th quartile of units of medication dispensed.

**Figure 2:**
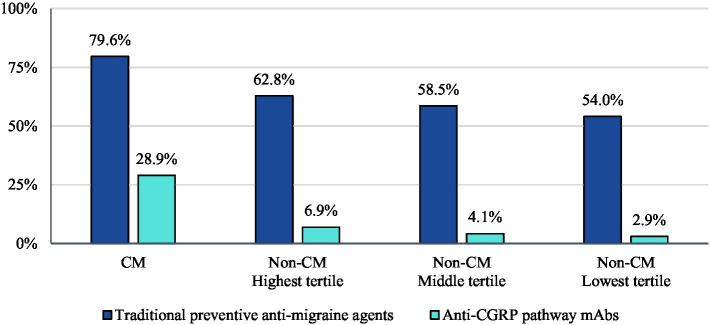
Proportion of patients with use of preventive migraine medications during the follow-up period.

In the 12-month follow-up period, CM patients had 70 units (mean) of AMSMs dispensed (median 54). The highest, middle, and lowest AMSM dispensed groups had mean values of 92 units (median: 81), 29 units (median: 27), and 10 units (median: 9), respectively (Figure 1). Assessment of AMSM use by type showed that triptan claims were higher in the follow-up period than baseline in nearly all CM (98.5%) and non-CM (99.8%) patients; non-triptan use in the follow-up period was similar to the baseline period (CM: 62.1%, non-CM: 45.1–47.6%). Utilization of traditional preventive medications was observed in a slightly higher proportion in CM patients than non-CM patients, with 79.6% having at least one claim in the follow-up period compared to 54.0–62.8% of non-CM patients (Figure 2). Of the preventive medication claims assessed, antidepressants and anticonvulsants were most frequently seen in both groups. Anti-CGRP pathway mAbs were dispensed for approximately a third of CM patients (28.9%) and for less than 10% of non-CM patients. Of the CM patients utilizing anti-CGRP pathway mAbs, the majority used erenumab; galcanezumab was the second most commonly used mAb. Of all non-CM patients utilizing anti-CGRP pathway mAbs, proportions were 17.8, 10.1, and 4.4% across erenumab, galcanezumab, and fremanezumab, respectively.

### 3.3 Provider visits in follow-up

**Figure 3:**
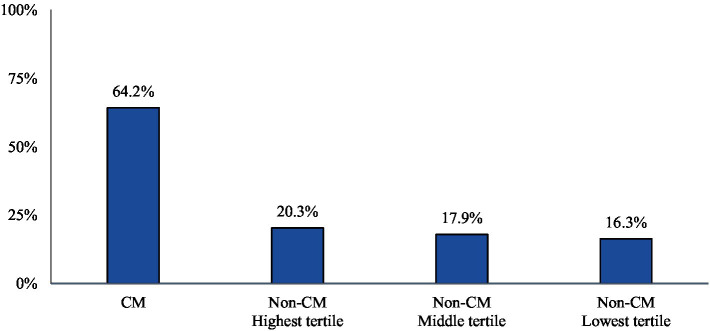
Proportion of patients with a claim indicating a visit to a headache specialist or neurologist during the study follow-up-period. Patients who qualify, may be more likely to be prescribed targeted therapy with anti-CGRP pathway mAbs over traditional preventative medicines.

The majority of CM patients had a visit to a headache specialist or neurologist (64.2%) in the follow-up period. The proportion of non-CM patients with a visit to a headache specialist or neurologist followed the trends of AMSM use (20.3, 17.9, and 16.3% of the highest, middle, and lowest tertiles, respectively) (Figure 3).

## 4 Discussion

MHDs—or specifically monthly migraine days (MMDs)—have served as the primary units of measure for a day during which the onset, continuation, or recurrence of a migraine headache is experienced (23, 24). MMDs have been criteria for the classification of migraine and, subsequently, the clinical management of migraine. Though informative, MMDs only partly reveal an individual’s experience with migraine and its associated symptoms. MMDs do not provide insight into migraine severity or impact, further underlining the arbitrary designation of migraine status in one of the two current categories.

The AHS Position Statement of 2024 notes that preventive management of migraines has changed. The AHS recommends prevention with anti-CGRP pathway treatment as the first line and further suggests that studies on non-specific treatments and anti-CGRP pathway therapies with respect to acute medication use and overuse led to this recommendation (22).

Since being first introduced in 2008, anti-CGRP pathway mAbs have been effective treatment options for migraine prevention. Their efficacy, safety, and tolerability have been demonstrated in clinical trials as well as in real-world studies. Our study set out to characterize the population of migraine patients in the US who would be eligible for preventive treatment with an anti-CGRP pathway mAb. Furthermore, the study aimed to better understand treatment patterns and the use of medication for migraine. In this assessment, a large number of patients were found to have significant utilization of AMSMs, reflected in the quantity of dispensed medications. We found that less than 10% had a diagnosis of CM, and a large majority of these CM patients had a prescription for a traditional non-specific preventive migraine medication in the year after their recorded diagnosis.

Within the non-CM population, the most informative findings emerged from the highest tertile subgroup. Although the majority (63%) of non-CM patients in the highest tertile were on preventive migraine medications, their use of AMSM was high (mean: 92 units; median: 81 units). In fact, the quantity of dispensed AMSM in this subgroup exceeded that of CM patients (mean: 70 units; median: 54 units), suggesting that the preventive treatment in the highest tertile non-CM patients is either ineffective or sub-optimal, as additional acute medications continue to be utilized to abate symptoms. Interestingly, the proportion of non-CM patients in the highest tertile who had use of anti-CGRP pathway mAbs for migraine prevention was not only notably lower than CM patients but comparable to non-CM patients in the middle and lowest tertiles, implying that the treatment approach for non-CM patients in the highest tertile is very similar to the other two non-CM tertiles, despite their disease burden being nearly as high as CM patients. These findings may be explained by the pattern of visits to a headache specialist or neurologist, who may more likely prescribe an anti-CGRP pathway mAb. The proportion of highest tertile non-CM patients who had a visit to a headache specialist or neurologist in the follow-up period is similar to the other two tertiles of non-CM patients. As noted, anti-CGRP pathway treatments are recommended by AHS as a first-line option for migraine prevention without the requirement of prior treatment failure with traditional preventives (22). This recommendation is based on vast clinical data, including improvements in acute migraine medication use, observational study outcomes, and real-world evidence (25).

The results from our study further highlight the unmet need for treatment with appropriate preventive medications and the persistent gap in management of patients without a diagnosis of CM who are dispensed high quantities of AMSM. Our findings raise the importance of migraine management under the care of a headache specialist or neurologist, and if not feasible, a healthcare provider trained in migraine management, for the subgroup of non-CM patients with high disease burden. Finally, although not an objective of the study, the findings support the heterogeneity of the non-CM population and the arbitrary designation of migraine classification based on MMDs.

Our study has certain limitations inherent to the utilization of administrative claims databases as data sources. For example, coding errors or incorrect documentation of medical conditions and medications may result in the misclassification of patients or an under/over estimation of medication use. Furthermore, evidence of prescription claims does not necessarily imply the consumption of medication by the patient, only that the prescription was filled. Any medication obtained outside of insurance coverage (i.e., free samples or discount cards from another country) cannot be captured and included in the analysis, potentially underestimating medication use. AMSMs are presented in dispensed units, which are drawn from outpatient pharmacy claims; while it is an accurate representation of medication on hand for the patient, we cannot assert that patients consumed all units of dispensed medication. A potential barrier for prevention with anti-CGRP pathway mAbs may be access, whether through health plan coverage or financial reasons; however, this was not assessed in this analysis. All patients in this analysis had continuous enrollment in a health plan; therefore, these results may not be generalizable to an uninsured population or a population that has an older average age. Another potential limitation, as with other studies using diagnostic codes, is whether the codes accurately reflect the patient’s condition. It is possible that CM might be underdiagnosed, perhaps due to fluctuations in the pattern of migraine attacks or behavioral/reporting variations in patient subpopulations. Hence, incorporating additional information, such as free text clinical notes, when available, is an important consideration in future research.

## 5 Conclusion

The results from this real-world study further confirmed the unmet need for treatment with appropriate preventive medication and a persistent gap in the management of patients without a CM diagnostic code who are dispensed high quantities of AMSM. Non-CM patients with similar disease burden to CM patients had patterns of migraine management similar to non-CM patients of lower disease burden (i.e., visits to a headache specialist or neurologist or use of anti-CGRP pathway mAb), acknowledging that we were unable to investigate whether health plan coverage had an impact on the aforementioned parameters and that CM could be underdiagnosed. Additional research using alternative real-world data sources that could potentially address the limitations of our study is required. Finally, our findings emphasize the ongoing discussion on the need to redefine migraine classification in order to more appropriately reflect the patient’s disease experience and burden.

## Data availability statement

The data analyzed in this study is subject to the following licenses/restrictions: HIPAA Restrictions. Requests to access these datasets should be directed to kevin.hawkins@iqvia.com.

## Ethics statement

Ethical approval was not required for the study involving humans in accordance with the local legislation and institutional requirements. Written informed consent to participate in this study was not required from the participants or the participants’ legal guardians/next of kin in accordance with the national legislation and the institutional requirements.

## Author contributions

AK: Writing – review & editing, Writing – original draft. JM: Writing – original draft, Writing – review & editing. SO: Writing – review & editing, Writing – original draft. FV: Writing – original draft, Writing – review & editing. MB: Writing – review & editing, Writing – original draft. KS: Writing – review & editing, Writing – original draft. KH: Writing – original draft, Writing – review & editing. C-CC: Writing – review & editing, Writing – original draft. AG: Writing – review & editing, Writing – original draft. ST: Writing – review & editing, Writing – original draft.

## References

[1] Estave PM Beeghly S Anderson R Margol C Shakir M George G. Learning the full impact of migraine through patient voices: a qualitative study. Headache. (2021) 61:1004–20. doi: 10.1111/head.14151, PMID: 34081779 PMC8428538

[2] Buse DC Fanning KM Reed ML Murray S Dumas PK Adams AM. Life with migraine: effects on relationships, career, and finances from the chronic migraine epidemiology and outcomes (CaMEO) study. Headache. (2019) 59:1286–99. doi: 10.1111/head.13613, PMID: 31407321 PMC6771487

[3] Walter K. What is migraine? JAMA. (2022) 327:93. doi: 10.1001/jama.2021.21857 34982118

[4] Tana C Raffaelli B Souza MNP de la Torre ER Massi DG Kisani N. Health equity, care access and quality in headache – part 1. J Headache Pain. (2024) 25:12. doi: 10.1186/s10194-024-01712-7 38281917 PMC10823691

[5] Lipton RB Nicholson RA Reed ML Araujo AB Jaffe DH Faries DE. Diagnosis, consultation, treatment, and impact of migraine in the US: results of the OVERCOME (US) study. Headache. (2022) 62:122–40. doi: 10.1111/head.14259, PMID: 35076091 PMC9305407

[6] Burch R Rizzoli P Loder E. The prevalence and impact of migraine and severe headache in the United States: updated age, sex, and socioeconomic‐specific estimates from government health surveys. Headache. (2021) 61:60–8. doi: 10.1111/head.14024 33349955

[7] Mannix S Skalicky A Buse DC Desai P Sapra S Ortmeier B. Measuring the impact of migraine for evaluating outcomes of preventive treatments for migraine headaches. Health Qual Life Outcomes. (2016) 14:143. doi: 10.1186/s12955-016-0542-3, PMID: 27716228 PMC5053168

[8] Gibbs SN Shah S Deshpande CG Bensink ME Broder MS Dumas PK. United States patients’ perspective of living with migraine: country-specific results from the global “my migraine voice” survey. Headache. (2020) 60:1351–64. doi: 10.1111/head.13829, PMID: 32369201 PMC7496834

[9] Harris L L’Italien G Kumar A Seelam P LaVallee C Coric V. Real-world assessment of the relationship between migraine-related disability and healthcare costs in the United States. Headache. (2022) 62:473–81. doi: 10.1111/head.14289, PMID: 35343590 PMC9313575

[10] Headache Classification Committee of the International Headache Society (IHS). The international classification of headache disorders, 3rd edition. Cephalalgia. (2018) 38:1–211. doi: 10.1177/0333102417738202, PMID: 29368949

[11] Buse DC Reed ML Fanning KM Bostic RC Lipton RB. Demographics, headache features, and comorbidity profiles in relation to headache frequency in people with migraine: results of the American migraine prevalence and prevention (AMPP) study. Headache. (2020) 60:2340–56. doi: 10.1111/head.13966, PMID: 33090481

[12] Ishii R Schwedt TJ Dumkrieger G Lalvani N Craven A Goadsby PJ. Chronic versus episodic migraine: the 15-day threshold does not adequately reflect substantial differences in disability across the full spectrum of headache frequency. Headache. (2021) 61:992–1003. doi: 10.1111/head.14154 34081791

[13] Ailani J Burch RC Robbins MS Board of Directors of the American Headache Society. The American headache society consensus statement: update on integrating new migraine treatments into clinical practice. Headache. (2021) 61:1021–39. doi: 10.1111/head.14153, PMID: 34160823

[14] Hepp Z Dodick DW Varon SF Chia J Matthew N Gillard P. Persistence and switching patterns of oral migraine prophylactic medications among patients with chronic migraine: a retrospective claims analysis. Cephalalgia. (2017) 37:470–85. doi: 10.1177/0333102416678382, PMID: 27837173 PMC5405847

[15] Raffaelli B Rubio-Beltran E Cho SJ De Icco R Labastida-Ramirez A Onan D. Health equity, care access and quality in headache – part 2, 167. J Headache Pain. (2023) 24. doi: 10.1186/s10194-023-01699-7, PMID: PMC10717448 38087219

[16] Katsarava Z Buse DC Manack AN Lipton RB. Defining the differences between episodic migraine and chronic migraine. Curr Pain Headache Rep. (2012) 16:86–92. doi: 10.1007/s11916-011-0233-z, PMID: 22083262 PMC3258393

[17] Bigal ME Serrano D Buse D Scher A Stewart WF Lipton RB. Acute migraine medications and evolution from episodic to chronic migraine: a longitudinal population-based study. Headache. (2008) 48:1157–68. doi: 10.1111/j.1526-4610.2008.01217.x 18808500

[18] Chiang CC Schwedt TJ Wang SJ Dodick DW. Treatment of medication-overuse headache: a systematic review. Cephalalgia. (2016) 36:371–86. doi: 10.1177/0333102415593088 26122645

[19] Schwedt TJ Hentz JG Sahai-Srivastava S Murinova N Spare NM Treppendahl C. Patient-centered treatment of chronic migraine with medication overuse: a prospective, randomized, pragmatic clinical trial. Neurology. (2022) 98:e1409–21. doi: 10.1212/WNL.0000000000200117, PMID: 35169011

[20] Blumenfeld AM Bloudek LM Becker WJ Buse DC Varon SF Maglinte GA. Patterns of use and reasons for discontinuation of prophylactic medications for episodic migraine and chronic migraine: results from the second international burden of migraine study (IBMS-II). Headache. (2013) 53:644–55. doi: 10.1111/head.12055 23458496

[21] Faust E Pivneva I Yang K Betts KA Ahmed Z Joshi S. Real-world treatment profiles, clinical outcomes, and healthcare resource utilization of patients with migraine prescribed Erenumab: a multicenter chart-review study of US headache centers. Neurol Ther. (2021) 10:293–306. doi: 10.1007/s40120-021-00245-4, PMID: 33856626 PMC8140045

[22] Charles AC Digre KB Goadsby PJ Robbins MS Hershey A American Headache Society. Calcitonin gene-related peptide-targeting therapies are a first-line option for the prevention of migraine: an American Headache Society position statement update. Headache. (2024) 64:333–41. doi: 10.1111/head.14692 38466028

[23] Silberstein SD Holland S Freitag F Dodick DW Argoff C Ashman E. Evidence-based guideline update: pharmacologic treatment for episodic migraine prevention in adults: Table 1. Neurology. (2012) 78:1337–45. doi: 10.1212/wnl.0b013e3182535d20, PMID: 22529202 PMC3335452

[24] Headache Classification Committee of the International Headache Society (IHS). The international classification of headache disorders, 3rd edition (beta version). Cephalalgia. (2013) 33:629–808. doi: 10.1177/0333102413485658 23771276

[25] Caronna E Gallardo VJ Egeo G Vázquez MM Castellanos CN Membrilla JA. Redefining migraine prevention: early treatment with anti-CGRP monoclonal antibodies enhances response in the real world. J Neurol Neurosurg Psychiatry. (2024):jnnp-2023-333295. doi: 10.1136/jnnp-2023-333295, PMID: 38777579

